# Radiofrequency thalamotomy for tremor produces focused and predictable lesions shown on magnetic resonance images

**DOI:** 10.1093/braincomms/fcad329

**Published:** 2023-11-30

**Authors:** Bryony K Ishihara, Michael G Hart, Thomas R Barrick, Franklyn A Howe, Francesca Morgante, Erlick A Pereira

**Affiliations:** Neurosciences Research Centre, Molecular and Clinical Sciences Research Institute, St George’s, University of London, London SW17 0RE, UK; Neurosciences Research Centre, Molecular and Clinical Sciences Research Institute, St George’s, University of London, London SW17 0RE, UK; Neurosciences Research Centre, Molecular and Clinical Sciences Research Institute, St George’s, University of London, London SW17 0RE, UK; Neurosciences Research Centre, Molecular and Clinical Sciences Research Institute, St George’s, University of London, London SW17 0RE, UK; Neurosciences Research Centre, Molecular and Clinical Sciences Research Institute, St George’s, University of London, London SW17 0RE, UK; Department of Experimental and Clinical Medicine, University of Messina, 98122 Messina, Italy; Neurosciences Research Centre, Molecular and Clinical Sciences Research Institute, St George’s, University of London, London SW17 0RE, UK

**Keywords:** thalamotomy, Parkinson’s disease, essential tremor, lesion, magnetic resonance imaging

## Abstract

Radiofrequency thalamotomy is a neurosurgical management option for medically-refractory tremor. In this observational study, we evaluate the MRI features of the resultant lesion, their temporal dynamics, and how they vary depending on surgical factors. We report on lesion characteristics including size and location, as well as how these vary over time and across different MRI sequences. Data from 12 patients (2 essential tremor, 10 Parkinson’s disease) who underwent unilateral radiofrequency thalamotomy for tremor were analysed. Lesion characteristics were compared across five structural sequences. Volumetric analysis of lesion features was performed at early (<5 weeks) and late (>5 months) timepoints by manual segmentation. Lesion location was determined after registration of lesions to standard space. All patients showed tremor improvement (clinical global impressions scale) postoperatively. Chronic side-effects included balance disturbances (*n* = 4) and worsening mobility due to parkinsonism progression (*n* = 1). Early lesion features including a necrotic core, cytotoxic oedema and perilesional oedema were best demarcated on T_2_-weighted sequences. Multiple lesions were associated with greater cytotoxic oedema compared with single lesions (T_2_-weighted mean volume: 537 ± 112 mm³ versus 302 ± 146 mm³, *P* = 0.028). Total lesion volume reduced on average by 90% between the early and late scans (T_2_-weighted mean volume: 918 ± 517 versus 75 ± 50 mm³, t = 3.592, *P* = 0.023, *n* = 5), with comparable volumes demonstrated at ∼6 months after surgery. Lesion volumes on susceptibility-weighted images were larger than those of T_2_-weighted images at later timepoints. Radiofrequency thalamotomy produces focused and predictable lesion imaging characteristics over time. T_2_-weighted scans distinguish between the early lesion core and oedema characteristics, while lesions may remain more visible on susceptibility-weighted images in the months following surgery. Scanning patients in the immediate postoperative period and then at 6 months is clinically meaningful for understanding the anatomical basis of the transient and permanent effects of thalamotomy.

## Teaser

Ishihara *et al*. report that early lesion features (necrotic core, cytotoxic and perilesional oedemas) following radiofrequency thalamotomy for movement disorders are best demarcated on T_2_-weighted MRI. Lesion volumes were significantly reduced by 6 months after surgery. Overall, MRI demonstrates that radiofrequency thalamotomy produces focused and predictable lesion characteristics over time.

## Graphical Abstract

**Graphical Abstract:**
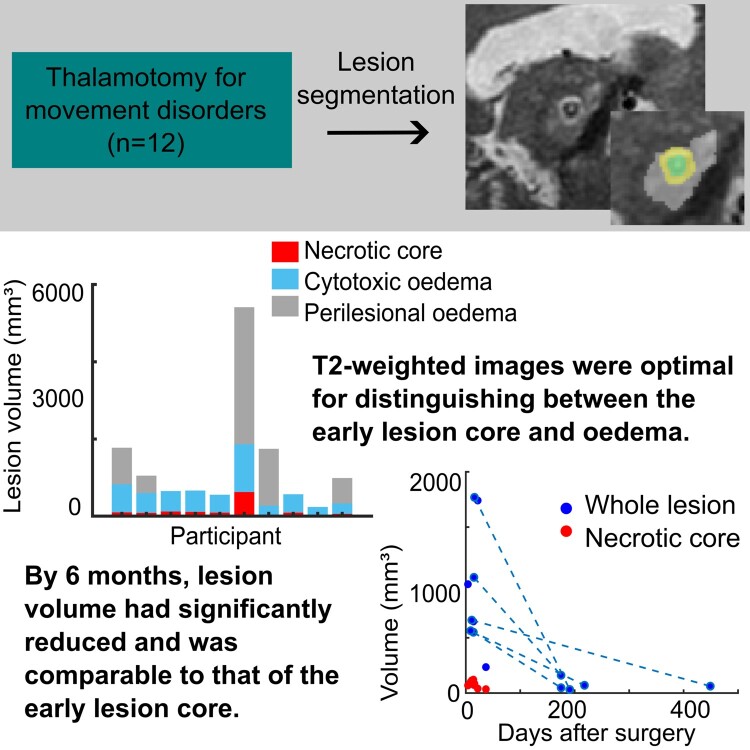

## Introduction

Tremor due to Parkinson’s disease and essential tremor is often inadequately managed with oral medication but responds well to neurosurgery.^1^ Unilateral radiofrequency (RF) thalamotomy is an effective treatment for medically-refractory tremor involving lesioning of the ventral intermediate (VIM) nucleus of the thalamus.^2,3^ While neurosurgical techniques such as deep brain stimulation (DBS) and magnetic resonance-guided focused ultrasound (MRgFUS) thalamotomy have become increasingly common in the treatment of movement disorders, RF thalamotomy remains an important therapeutic option owing to its simplicity, effectiveness, wealth of supporting clinical experience and favourable health economics profile.^4-6^

Despite this experience, there are few studies on the MRI features of thalamic lesions created by RF, and to our knowledge, there has been no comparison of different imaging sequences in defining RF thalamotomy lesions in humans. This study evaluated RF thalamotomy lesions using multiple MRI sequences to understand lesion characteristics and temporal dynamics, and how they varied depending on surgical factors. Our primary aims were to inform postoperative MR imaging protocols for future prospective studies, and to better understand lesion characteristics for comparison between different lesioning modalities e.g. MRgFUS.

## Materials and methods

This study was approved by the National Research Ethics Service Local Research Ethics Committee (IRAS: 259146).

### Participants

A prospective series of patients between 2017 and 2022 discussed in the movement disorders multidisciplinary team (MDT) at St George’s Hospital, London and deemed eligible for RF thalamotomy were identified retrospectively (*n* = 14). Patient selection was based upon recognized criteria for DBS in movement disorders. Additionally, a radiofrequency lesion was favoured when the symptom impact was predominantly unilateral and there were extenuating medical co-morbidities that made general anaesthesia or implantation of a device less appealing. Finally, patient choice was a key criterion particularly if an immediate effect or reduction in visits for programming were meaningful. Two of these patients did not receive RF thalamotomy after testing and were therefore excluded.

### Surgical procedure

Stereotactic surgery was performed using a Cosman–Roberts–Wells (CRW) stereotactic frame (Integra, USA) under local anaesthesia while patients were fully awake. Targeting was performed using Renishaw Neuronspire™ (Renishaw plc, UK). Indirect coordinates 11–15 mm lateral and 4 mm posterior to the mid-commissural point (MCP) ^7^ were refined to ensure that the target was ∼10 mm from the lateral border of the third ventricle on T_2_-weighted imaging and 2–3 mm from the medial internal capsule on fast grey matter acquisition T1 inversion recovery (FGATIR) at the level of the MCP (Z depth = 0).^8^ A 2 mm diameter reusable radiofrequency thermocoagulation electrode with a 2 mm uninsulated tip (TC-16-2-250-D electrode and Cosman G4 generator; Boston Scientific Corporation, Marlborough, MA, USA) was inserted after twistdrill craniotomy.

Neurological assessment was performed in the awake patient by a board-certified consultant neurologist. Macrostimulation utilized the G4™ RF Generator (Boston Scientific Corporation, Valencia, CA, USA). Parameters included inhibitory testing at a frequency of 50 Hz and pulse width of 0.5 ms to identify voltage thresholds for capsular and sensory effects. Motor stimulation was performed at 2 Hz and 0.5 ms. Depth was adjusted with a CRW manual microdrive (Integra, USA) to identify tremor improvement based on clinical assessment and side-effect thresholds. Lesions were made by heating the electrode to 80°C. Lesioning parameters, specifically number of lesions and their duration, were determined based on the underlying symptoms, clinical response and physiological effects *viz a viz* motor and sensory impairment. For example, with an early response to less severe symptoms, our minimum duration was 30 s, while in another case with more severe tremor, three lesions were used for a total of 210 s as they were well tolerated without side-effects. If multiple lesions were considered, our preference was to extend along the existing tract in the first instance, which was typically planned to allow safe progression into the zona incerta.

### MRI acquisition

MR images were acquired on a 3.0 tesla (3 T) scanner with a 32-channel head coil (Philips dual Tx Acheiva, St George’s Hospital). The clinical imaging procedure was to scan patients preoperatively, and at 2 weeks and 6 months postoperatively. The MR imaging protocol included the following sequences: magnetization prepared rapid gradient echo (MPRAGE, T_1_-weighted, TR = 6.8 ms, TE = 3.1 ms, TI = 819 ms, flip angle = 8°, 1 mm isotropic spatial resolution); T_2_-weighted (TR = 2200 ms, TE = 258 ms, flip angle = 90°, spatial resolution = 0.65 × 0.65 × 0.8 mm); fluid attenuated inversion recovery (FLAIR, TR = 6000 ms, TE = 274 ms, IR = 2200 ms, flip angle = 40°, spatial resolution = 0.98 × 0.98 × 1 mm); susceptibility-weighted imaging (SWI, TR = 22 ms, TE = 32 ms, flip angle = 15°, spatial resolution = 0.49 × 0.49 × 1 mm) and fast grey matter acquisition T1 inversion recovery (FGATIR, TR = 7.5 ms, TE = 3.5 ms, flip angle = 8°, spatial resolution = 0.625 × 0.625 × 1 mm).

### Clinical outcome

Patients were evaluated by a neurologist using the clinical global impressions (CGI) scale (measure of clinical improvement on a scale of 1—very much improved, to 7—very much worse) in postoperative Day 0 and then at 3 and 12 months postoperatively. In-depth structured assessments at multiple timepoints were not routinely performed in accordance with our aim of minimizing logistical burdens in an often physically impaired patient cohort.

### Lesion analysis

Imaging analysis was performed using FSL (FMRIB Software Library v6.0).^9,10^

#### Expanding spherical signal intensity analysis

**Figure 1:**
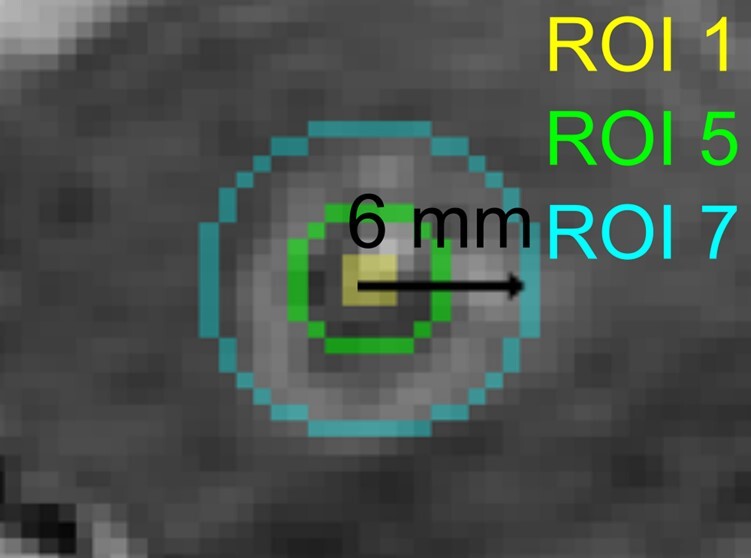
**Example spherical regions of interest (ROIs) for one patient in the sagittal view.** ROI 1 = 0.5 mm radius sphere surrounding centre of lesion coordinate, ROI 5 = spherical area 2.5–3 mm from centre of lesion, ROI 11 = spherical area 5.5–6 mm from centre of lesion.

Lesion appearances on different MR sequences were compared on a single patient by analysing their voxel-wise MRI signal intensities in native space. For each sequence (MPRAGE, T_2_-weighted, FLAIR, SWI and FGATIR), a seed coordinate was determined at the centre of the lesion. From this point, 11 spherical regions of interest (ROIs) were created with increasing radiuses (1–6 mm in increments of 0.5 mm). For each ROI, the previous ROI (radius smaller by 0.5 mm) was subtracted, and the mean signal intensity extracted (Fig. 1). Signal intensity was then normalized by dividing by the mean intensity of the surrounding intact tissue of the thalamus. This analysis was performed on an example patient exhibiting a typical lesion appearance. The average across patients was not taken due to differences in lesion volume.

#### Signal intensity change analysis

The FIRST automatic segmentation tool was used to obtain pre and postoperative thalamus segmentations on MPRAGE images.^11^ MPRAGE images were registered to T_2_-weighted for both pre and postoperative timepoints using FLIRT.^12,13^ A lesion cost function weighting was used for postoperative image registration. These transformations were then applied to the FIRST thalamus segmentations to align them to T_2_-weighted images. Histogram values were extracted for *n* = 10 bins (0–2000 intensity range) and averaged across seven participants (those that had the required images) to produce histograms showing T_2_-weighted signal intensity changes in the thalamus following thalamotomy.

#### Lesion volumetry

Volumetric lesion segmentation was performed manually on all slices with the lesion using FSLeyes.^14^ For early scans, FLAIR and T_2_-weighted lesions were segmented into three concentric regions previously described by Wintermark *et al*.:^15^ (i) the necrotic lesion core (hypointense); (ii) cytotoxic oedema (markedly hyperintense); and (iii) the surrounding perilesional oedema (moderately hyperintense). At the later scans, these zones were not present and so the entire visible lesion was segmented (lesions were only visible on T_2_-weighted, FLAIR and SWI). The volumes of each lesion mask were obtained in mm³.

#### Lesion localization

Lesion location was determined following brain extraction (using BET)^16^ by a two-stage registration of (i) T_2_-weighted images to the corresponding pre-procedural T_1_-weighted image (linear registration using FLIRT with a lesion cost function weighting);^12,13^ and (ii) registration of the T_1_-weighted MPRAGE image to the Montreal Neurological Institute (MNI) template (non-linear registration using FNIRT).^17^ The transforms obtained from registration were applied to the lesion segmentations using nearest neighbour interpolation (final image resolution was 1 mm isotropic). Right-sided lesions were flipped at the mid-sagittal plane to allow comparison of the anatomical location of all lesions. Lesion location was determined for each patient by calculating percentage overlap of lesion voxels with a VIM atlas (distal atlas, Lead-DBS)^18^ and FGATIR hypointensity template (template of a recently identified hypointensity on FGATIR at the base of the thalamus).^19^

### Statistical analysis

Statistical analysis was performed using IBM SPSS Statistics (Version 28.0.0.0). Two repeated measures ANOVAs with pairwise comparisons (Bonferroni adjusted) were applied to the volumetric data. One ANOVA examined the effect of sequence and lesion number on early lesion zone volumes for the full cohort with T_2_ and FLAIR data (*n* = 10), while the other ANOVA was applied to the cohort with two postoperative scans (*n* = 5) to analyse the effect of sequence and time on total lesion volume. All data are presented as a mean ± standard deviation (SD).

## Results

### All patients had clinically meaningful tremor improvement postoperatively

Participants were 12 patients (mean age of 74.6 ± 3.4 years; 4 females) who underwent unilateral RF thalamotomy at St George’s Hospital, London, from 2017 to 2022. All patients had a preoperative diagnosis of medication-refractory Parkinson's disease (*n* = 10) or essential tremor (*n* = 2).

All 12 patients had clinically meaningful tremor improvement postoperatively, as assessed by a neurologist (CGI score of between 1 and 3). Five patients presented with persistent adverse effects that included four instances of balance disturbances and a case of worsening mobility due to parkinsonism progression (Table 1). No repeat procedures were performed.

**Table 1:** Patient characteristics and clinical outcome measured using the clinical global impressions (CGI) scale

Patient	Diagnosis	Patient CGI	Clinician CGI	Number of lesions	Early side-effects	Persistent side-effects
01	Parkinson's disease	1	1	2	None	None
02	Parkinson's disease	1	1	2	None	None
03	Essential tremor	2	2	1	Dysarthria and mild gait disturbances	None
04	Parkinson's disease	1	1	2	Mild worsening of previously existing balance disturbances and mild dysarthria	Mild balance disturbances
05	Parkinson's disease	2	2	2	Balance disturbances	Balance disturbances
06	Parkinson's disease	1	1	2	Delirium, dysarthria, balance disturbances	Balance disturbances
07	Parkinson's disease	2	2	3	Mild balance disturbances, mild dysarthria and subtle left side dysmetria	Mild balance disturbances
08	Parkinson's disease	1	1	1	None	None
09	Parkinson's disease	2	2	2	Delirium	None
10	Essential tremor	1	1	1	Mild worsening of previously existing balance disturbances	None
11	Parkinson's disease	3	3	1	Delirium	Worsening of mobility due to parkinsonism progression
12	Parkinson's disease	1	1	1	None	None

1: very much improved; 2: much improved; 3: minimally improved; 4: no change; 5: minimally worse; 6: much worse; 7: very much worse.

### Lesion characteristics and signal intensity varied by MRI sequence

All 12 patients had a preoperative scan and a postoperative scan between 4 and 37 days following surgery (mean of 16 days). Five of these patients had an additional scan ∼5 months to a year following thalamotomy (∼8 months postoperatively on average).

**Figure 2:**
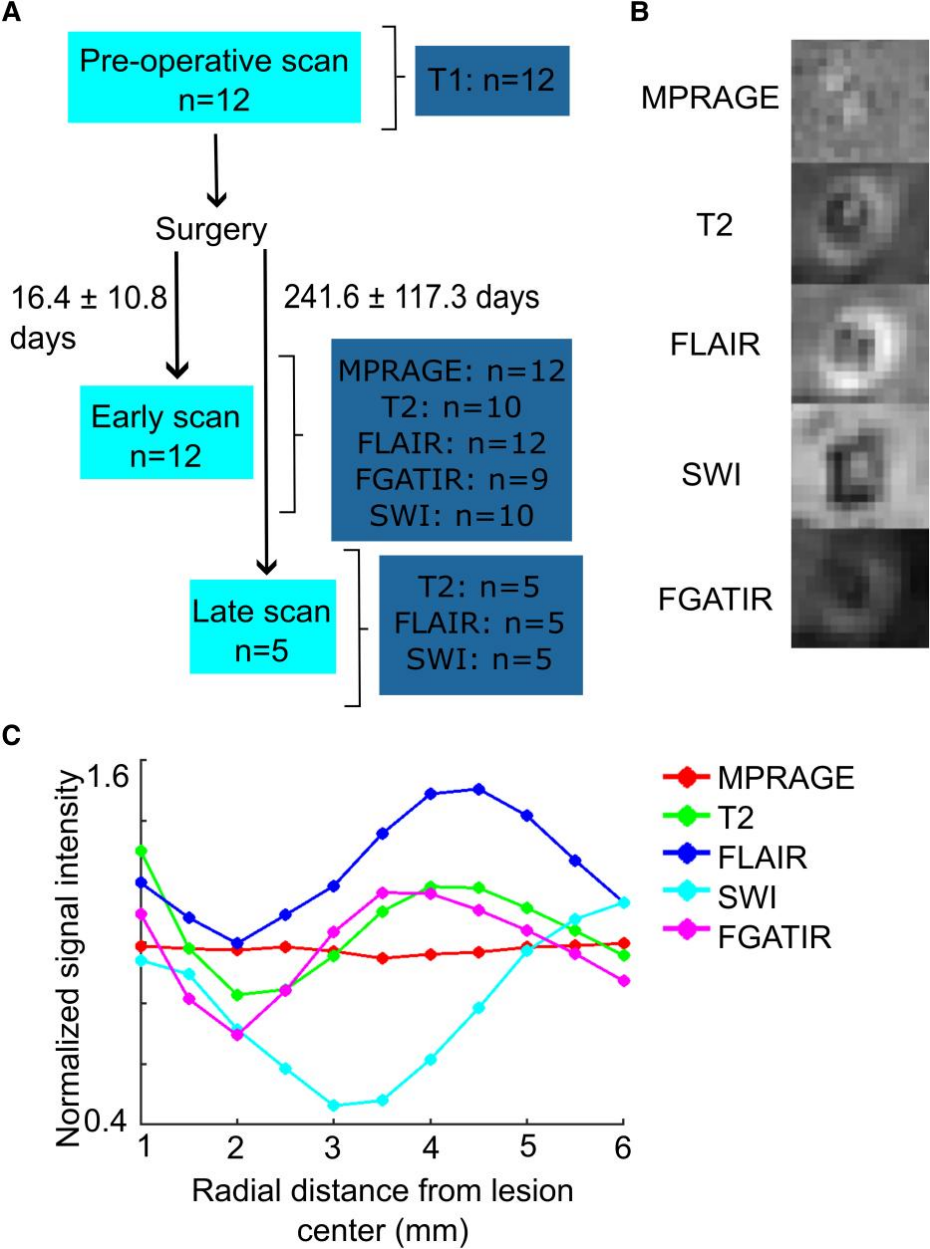
**(A) Flowchart of scanning procedure.** (**B**) Example lesion from one patient, visualized early (11 days after surgery) on MPRAGE, T_2_-weighted, FLAIR, SWI and FGATIR. Images are co-registered to MPRAGE. (**C**) Signal intensity graph corresponding to the MR images depicted in **B**. Data points show the mean signal intensity of spherical areas surrounding a seed coordinate at the centre of the lesion (see ‘Materials and methods’). Signal intensity was normalized by dividing by the mean intensity of the surrounding intact tissue of the thalamus.

The postoperative MR images (MPRAGE, T_2_-weighted, FLAIR, FGATIR and SWI sequences) of 12 patients who underwent thalamotomy for tremor were analysed. Lesions were incompletely visualized on MPRAGE whereas T_2_-weighted, FLAIR and FGATIR images showed a hypointense necrotic core surrounded by a region of hyperintense oedema (see Fig. 2B for example). A small sphere of hyperintensity could also be seen at the centre of the necrotic core in the majority of patients (7/10 on T_2_-weighted images). T_2_-weighted and FLAIR scans had a clear differentiation of the core versus oedema (Fig. 2C: signal intensity differences of 0.35 and 0.51, respectively, in the example lesion), as well as the oedema from surrounding tissue. In contrast, FGATIR did not clearly distinguish between oedema and healthy tissue (see Fig. 2B).

Presentation of the lesion centre on SWI was typically isointense compared to the surrounding thalamus, with a well-defined hypointense edge. The marked hypointensity on SWI images corresponded to the area surrounding the necrotic core on other sequences (see Fig. 2C at 3–4 mm radius from the centre) and allowed for a clear segmentation of the lesion.

### T_2_-weighted images showed a clear demarcation of lesion features of necrotic core, cytotoxic oedema and perilesional oedema

**Figure 3:**
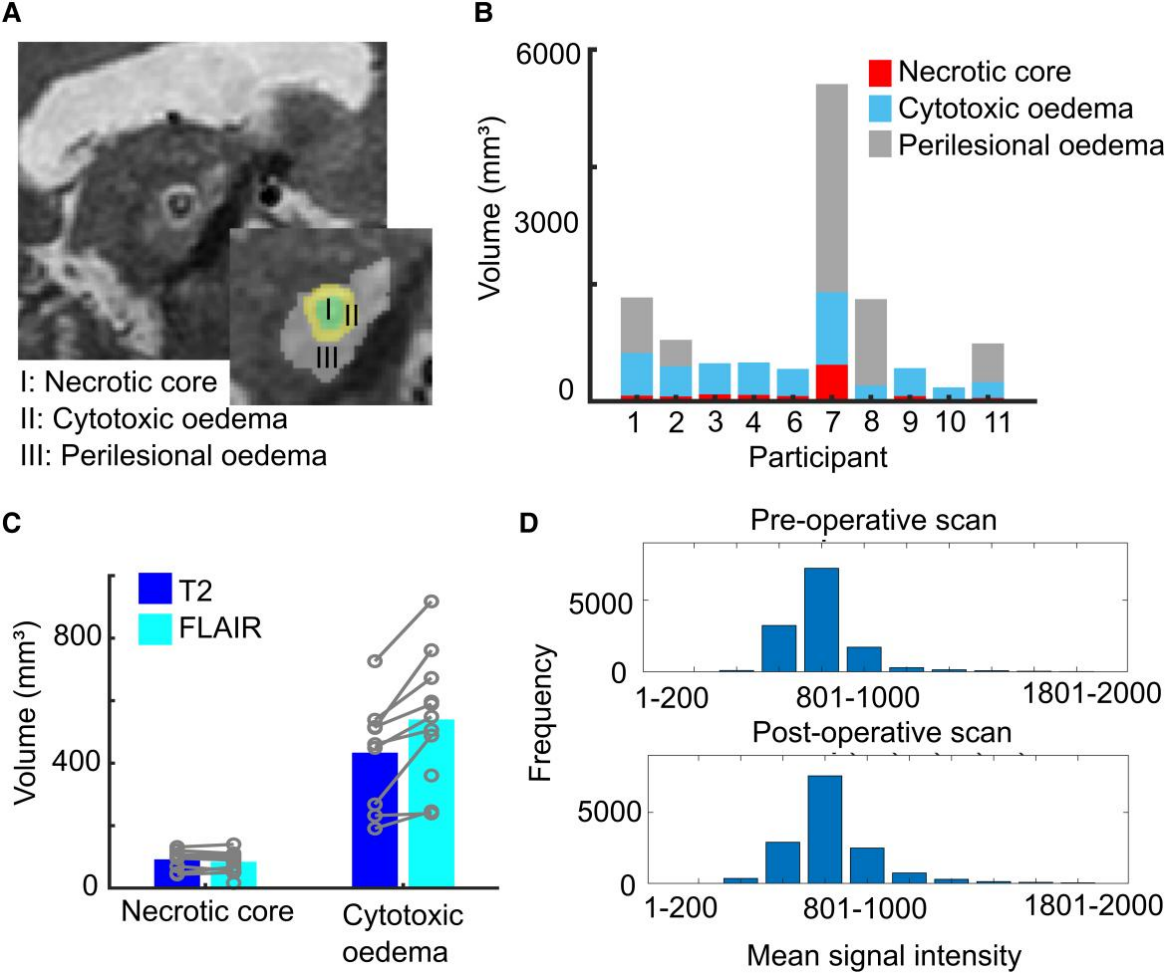
**(A) Example T_2_-weighted lesion showing volumetric segmentations of lesion zones (necrotic core: I; cytotoxic oedema: II; perilesional oedema: III) in the sagittal view.** (**B**) Volumes of lesion zones (necrotic core, cytotoxic oedema and perilesional oedema) on T_2_-weighted images at the first postoperative scan for each participant (*n* = 10). (**C**) Comparison of T_2_-weighted and FLAIR lesion zone volumes. An ANOVA showed that necrotic core volumes were not statistically different between T_2_ and FLAIR (*P* = 0.394, F = 0.843) whereas cytotoxic oedema differed (*P* = 0.005, F = 18.337). (**D**) Histograms showing changes (pre versus postoperative scans) to the mean signal intensity (*n* = 7) of the thalamus on T_2_-weighted images as a result of the lesion.

Four out of 12 participants presented with two levels of oedema visible on T_2_-weighted and FLAIR images (Fig. 3B); a strong hyperintense region of cytotoxic oedema and a moderately hyperintense region of perilesional oedema (see Fig. 3A for example). This has been previously described^15^ and was optimally demarcated on T_2_-weighted images. Nevertheless, the changes in signal between the pre and postoperative scans (Fig. 3D) do not show a discrete cut off and hence lesions required manual rather than automatic segmentation.

We performed a volumetric analysis of T_2_-weighted lesion features by manual segmentation. Volume of the necrotic core was consistent across participants (mean of 90 ± 31 mm³), while cytotoxic oedema demonstrated higher variance (mean of 433 ± 172 mm³). For patients that presented with perilesional oedema (*n* = 5), its volume tended to be greater than for cytotoxic oedema and varied more considerably between participants (mean of 2190 ± 1837 mm³). Neither the presence of perilesional oedema nor its volume was associated with postoperative complications (three out of five participants with perilesional oedema did not have chronic side-effects). One participant who received three lesions presented with a significantly greater total lesion volume compared to the other participants (5408 mm³ versus mean of 911 ± 535 mm³) and was excluded from further group level analysis. Clinically, this larger volume was associated with ‘much improved’ tremor but mild balance disturbances (Table 1, Participant 07).

Comparison of T_2_-weighted and FLAIR lesion volumes showed that while the volumes of the lesion core were consistent across T_2_-weighted and FLAIR images (*P* = 0.394), FLAIR had a larger area of hyperintense cytotoxic oedema (T_2_-weighted mean = 433 ± 172 mm³; FLAIR mean = 586 ± 221 mm³, *P* = 0.005). This greater hyperintensity on FLAIR may have contributed to the difficulty in distinguishing between the two levels of oedema using this sequence.

### Early lesion cores were consistently located in the VIM and overlapped with an FGATIR hypointensity template

**Figure 4:**
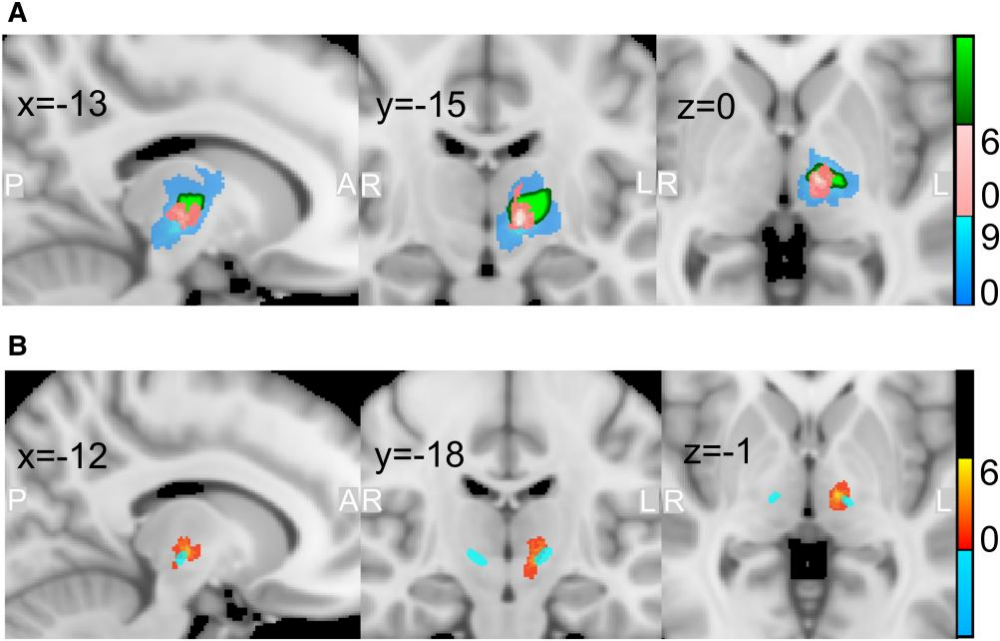
**Lesion location heatmap in MNI space (slice locations shown in mm, colour bar indicates the relationship between colour and *n*).** (**A**), The whole lesion (blue) and necrotic core (pink) segmentations from T_2_-weighted images (*n* = 9) were registered to the MNI template and overlayed. The VIM of the thalamus is indicated in green (distal medium atlas).^18^ (**B**) Overlap of T_2_-weighted necrotic core segmentations (red, *n* = 9) with an FGATIR hypointensity template^19^ (blue) in MNI space.

Lesion necrotic cores were well-localized to the VIM nucleus of the thalamus, with cytotoxic and perilesional oedemas expanding out of this area (Fig. 4A). Seven out of nine lesion necrotic cores overlapped with the VIM atlas with on average, 45.2% of core voxels showing an overlap.

Eight out of 9 lesion necrotic core segmentations overlapped to some degree with the FGATIR hypointensity template (mean overlap: 7.7 ± 6.0% of lesion voxels).

### Multiple lesions were associated with more cytotoxic oedema compared to single lesions

Patients received either a single lesion (*n* = 5) or multiple lesions (*n* = 7) according to intra-operative findings, with a cumulative heating time of 30–210 s. After exclusion of the outlier and participants with no T_2_-weighted image, there were four participants with single lesions and five with multiple lesions.

**Figure 5:**
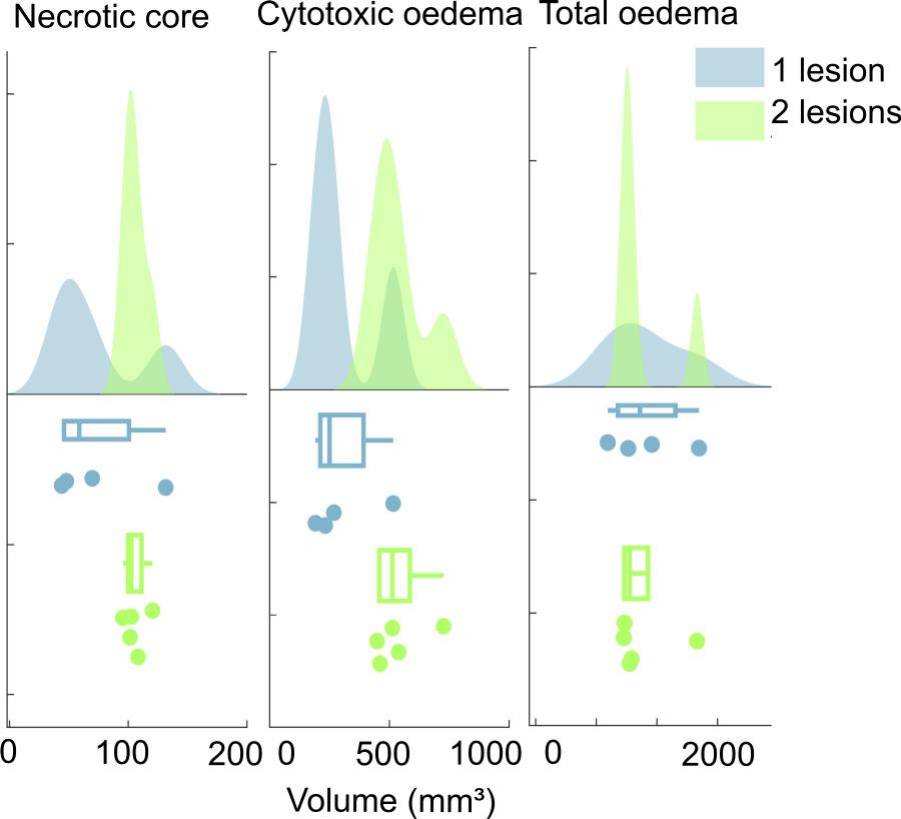
**Raincloud plot^20^ showing volume differences of lesion features (from T_2_-weighted images) between patients with one versus two lesions made.** An ANOVA with pairwise comparisons showed statistical difference values of *P* = 0.122 (F = 3.092) for necrotic core, *P* = 0.028 (F = 7.579) for cytotoxic oedema and *P* = 0.969 (F = 0.002) for total oedema.

Size of the necrotic core and total oedema volume, which included the perilesional oedema for those that had it, did not significantly vary by lesion number (*P* = 0.122 and *P* = 0.969, respectively). Multiple lesions were associated with greater cytotoxic oedema compared to single lesions. This difference was statistically significant, though should be interpreted with caution due to the low sample size (T_2_-weighted mean: 537 ± 112 mm³ versus 302 ± 146 mm³, *P* = 0.028) (Fig. 5).

### Lesion volume reduced significantly over time and appeared to stabilize at ∼6 months post-procedure

**Figure 6:**
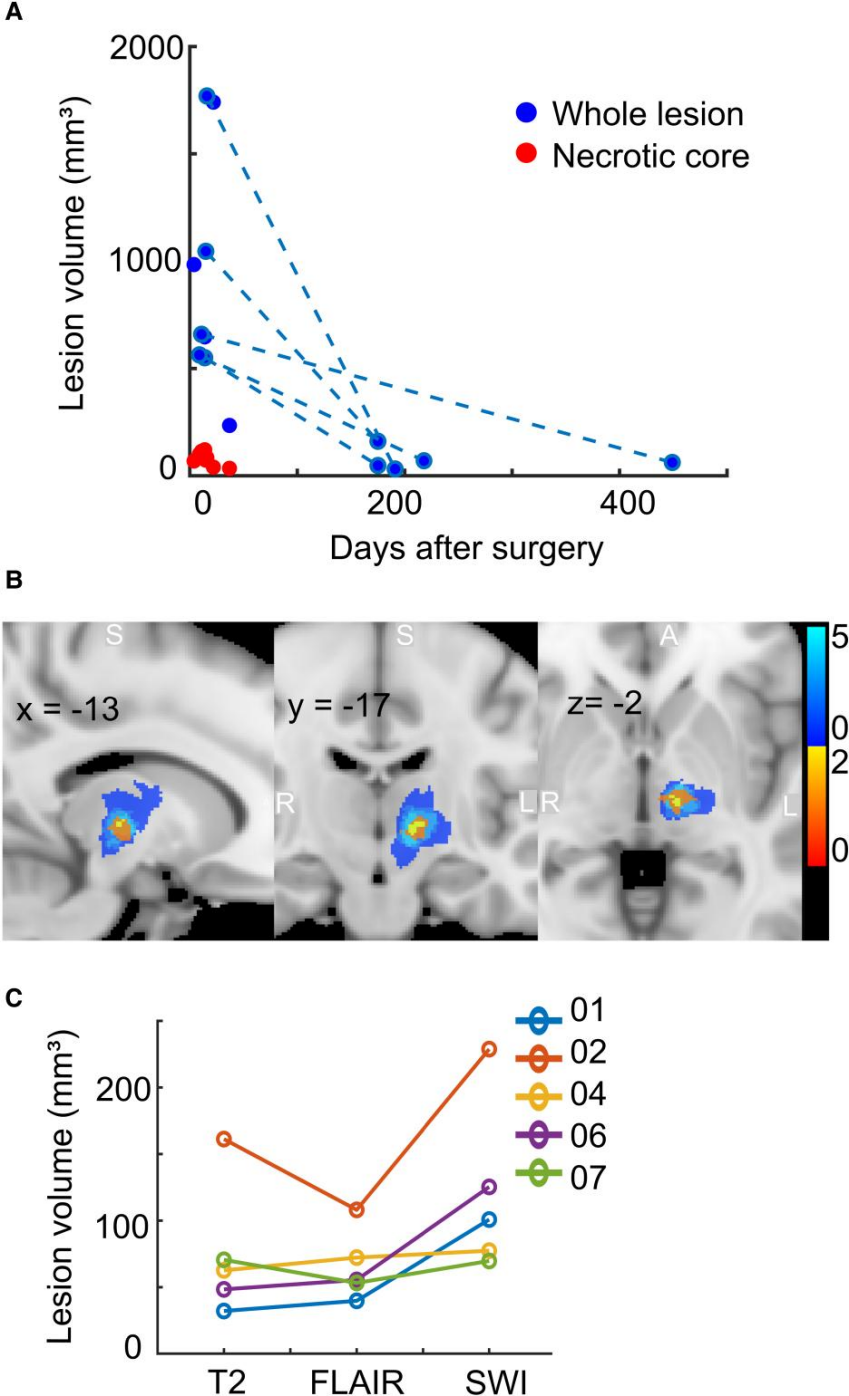
**(A) Graph of T_2_-weighted lesion volume reduction over time. Whole lesion volumes for each scan are plotted. Volumes at ‘early’ and ‘late’ timepoints are joined for patients who underwent two postoperative scans (*n* = 5).** An ANOVA with pairwise comparisons showed a statistical difference between ‘early’ and ‘late’ scans (*P* = 0.023, F = 12.9). Lesion core volumes at the early timepoint are plotted in red. (**B**) Change in lesion location between first (blue) and second (red-yellow) postoperative scans for five patients overlayed in MNI space. Slice location shown in mm. Colour bar indicates the relationship between colour and *n*. (**C**) Comparison of T_2_-weighted, FLAIR and SWI lesion volumes at second postoperative scan (*n* = 5, each participant in a different colour). SWI volumes were larger than T_2_-weighted, but not statistically different (*P* = 0.078, F = 14.005, ANOVA with pairwise comparisons).

Lesion volumes significantly reduced over time and apparently stabilized at ∼6 months post-procedure, as indicated by a reduction in volume variance between patients at early and late scans (50 mm³ versus 535 mm³ SD, Fig. 6A). For those patients who underwent multiple postoperative scans (*n* = 5), total lesion volume reduced on average by 90% between the earlier (12 ± 3 days post-surgery) and later (254 ± 131 days post-surgery) timepoints (mean of 918 ± 517 versus 75 ± 50 mm³, *P* = 0.023). The volume and location of lesions after 5 months were comparable to those of the lesion core at earlier timepoints, indicating a resolution of oedema over time while the necrotic core remained (Fig. 6A and B).

Figure 6C shows a comparison of lesion volumes from different sequence segmentations at the later timepoint. Lesions were not discernable on MPRAGE and FGATIR sequences after 5 months post-procedure. T_2_-weighted and FLAIR lesion volumes were similar (T_2_-weighted mean of 75 ± 50 mm³ versus 66 ± 26 mm³ for FLAIR, *P* = 1.000) whereas SWI segmentations were larger than those of corresponding T_2_-weighted images (SWI mean = 123 ± 62 mm³, Bonferroni corrected *P*-value of 0.078). However, statistically, lesion volume did not significantly differ by sequence.

## Discussion

We investigated the imaging characteristics of RF thalamotomy with the aim to better understand how lesion features are associated with time, surgical factors and specific MRI sequences. Improving understanding of MRI lesion characteristics following thalamotomy will inform postoperative imaging and follow-up to improve clinical outcomes and better understand lesion physiology.

### Early scan

The typical early (<5 weeks post-procedure) MR lesion characteristics present in T_2_-weighted, FLAIR and FGATIR sequences following RF thalamotomy for tremor were a hypointense necrotic core surrounded by a hyperintense region of cytotoxic oedema. In 5 out of 12 participants, a moderately hyperintense area of perilesional oedema was also visible on T_2_-weighted and FLAIR sequences. These MR characteristics of three concentric lesion zones are consistent with those previously reported for both RF and MRgFUS thalamotomy.^15,21,22^ It is notable that we also observed a small region of hyperintensity within the necrotic that is not accounted for in the volumetric analysis, and is most likely cystic in nature.

Regarding lesion location, in line with data demonstrating good spatial accuracy of stereotactic thalamotomy techniques, our findings showed good localization of lesions to the VIM of the thalamus.^4,21,23,24^ In addition, most lesion necrotic cores (8/9) showed some overlap with an atlas of a recently identified FGATIR hypointensity, thought to represent the dentatorubrothalamic tract (DRT).^19^ Recent evidence suggests that disruption of the DRT is associated with tremor improvement.^25,26^ Our finding further validates the potential of the FGATIR hypointensity as a visual marker for direct surgical targeting in thalamotomy for tremor.^19^

In contrast to previous reports following MRgFUS,^15,21^ perilesional oedema was not observed across all of our participants. Given that perilesional oedema significantly increases the total lesion volume and is associated with postoperative complications,^21^ our findings indicate that RF offers a more focused and predictable lesioning compared to MRgFUS, which would be expected to result in fewer postoperative deficits. In support of this, previously reported lesion sizes are smaller for RF^3^ compared to MRgFUS.^27^ The possible difference in predictability of lesions between these techniques may be related to the increased number of variables, e.g. skull density ratio, which affect the resultant lesion when using ultrasound.^28,29^ Alternatively, as 10 out of 12 participants had their first scan more than 1 week following surgery when perilesional oedema has been reported to be resolved,^5,15^ our participants may in fact have developed perilesional oedema that had dissipated by the time of their first scan. Therefore, a direct comparison of MRgFUS and RF thalamotomy is needed for better understanding of the surgical factors associated with development of perilesional oedema. This may help to reduce postoperative complications in the future.

Comparison of our volumetric findings with those of previous studies is difficult due to the variability in measurement technique and time of scan. However, lesion volumes appeared to be within normal ranges described by previous literature.^21-23^ It has been previously shown that the number of RF lesions made correlates positively with lesion volume.^30^ Our results indicate that this may be more specifically related to a greater volume of cytotoxic oedema development following multiple lesion creation. While participants with two compared to one lesion tended to have a larger cytotoxic oedema volume, we found no association between lesion number and volume of the necrotic core or perilesional oedema.

Early lesion characteristics were best demarcated on T_2_-weighted images, which showed a clearer distinction between cytotoxic and perilesional oedemas compared to FLAIR. The combination of T_1_- and T_2_-weighting in FLAIR imaging, as well as its lower spatial resolution, may have contributed to the observed differences in boundaries between these sequences. Despite the visible distinction of oedemas in T_2_-weighted images, it is notable that the lack of a discrete difference in signal between pre and postoperative T_2_-weighted scans suggests that lesion segmentation currently is best appreciated with manual segmentation. While lesions were well defined on SWI images by a strong hypointense edge that allowed for a clear lesion segmentation, this sequence may be less useful in the early stages following thalamotomy when the presence of oedema, not visible on SWI, is clinically relevant for explaining some transient postoperative side-effects.^21^

### Late scan

Lesions significantly reduced in volume over time and appeared to stabilize in size at ∼6 months after surgery. This is similar to a previous approximation of 7 months for stabilization of RF thalamotomy lesions.^22^ Lesion size at the second postoperative scan was comparable to that of the necrotic core at the first scan, suggesting that the reduction in volume over time was a resolution of the associated oedema. Overall, our findings suggest that scanning patients at 6 months post-procedure is reasonable for understanding the permanent effects of thalamotomy.

In contrast to previous findings for MRgFUS, reporting that lesions are often not discernible on T_2_-weighted images after 6 months,^29,31^ we found a visible T_2_-weighted lesion on all five patients who underwent a second postoperative scan (>5 months after surgery). However, SWI lesion segmentations were larger than those of T_2_-weighted (Fig. 6C), suggesting that this sequence may be more optimal for visualizing lesions at later timepoints. The better visibility of lesions on SWI is likely due to its sensitivity in detecting the presence of paramagnetic compounds such as hemosiderin remnants.^32^ In support of this, it has been demonstrated that SWI lesions remain discernible after MRgFUS when the same lesions are no longer visible on T_2_-weighted images.^31^ Given the association between shrinking of lesions over time and relapse of tremor,^31,33^ tracking SWI lesion volume and discernability may be useful for informing the need for repeat unilateral treatment. Indeed, this relationship may not be captured on T_2_-weighted images where patients have shown sustained clinical efficacy despite the absence of a T_2_-visible lesion.^5^ However, it is notable that lesions appear larger on SWI due to blooming artefacts.^34^

### Limitations

Our interpretation is limited by the fact that we grouped images acquired at different timepoints for analysis of early (4–37 days post-procedure) and late scans (175–449 days post-procedure). We did not seek to relate MRI lesion characteristics to clinical outcome in this study. However, we are standardizing our clinical follow-up as part of a multi-centre registry study (RAPID-CNS, Boston Scientific) that would enable such analysis in future. Regarding clinical data, we are planning to use the Parkinson’s Kinetigraph (PKG, Global Kinetics Corporation™) to offer a quantitative appraisal of symptom benefit while minimizing inconvenience and logistics.^35^ With regard to adverse events, while the reported rates are not low, this is most likely a feature of our assessments being undertaken by an independent neurologist and reported in a manner to maximize sensitivity. Furthermore, the severity of the impairments was often mild and functionally had minimal sequelae. Additionally, the majority of these effects readily resolved, and indeed effects were almost exclusively mild balance disturbances that would be difficult to discern from the effects of underlying disease progression. Finally, inherent differences in spatial resolution between sequences will likely affect delineation of the lesion, impacting on direct comparisons between sequences.

## Conclusions

RF thalamotomy produces focused and predictable lesion imaging characteristics, with lesion volume significantly reducing over time and apparently stabilizing at ∼6 months post-procedure. Therefore, scanning patients in the immediate postoperative period and then at 6 months is clinically relevant for understanding the transient and permanent effects of thalamotomy. T_2_-weighted images are useful for demarcation of early lesion features including oedema, while SWI may be more useful for visualizing lesions at later timepoints when discernability reduces on other sequences. Lesioning has experienced a renaissance driven by MRgFUS and the recognition of its relative merits *viz a viz* DBS. These data highlight the forgotten potential of radiofrequency lesioning, provide a valuable contribution to understanding the pathophysiology of brain lesions and are a noteworthy resource with which to compare the MRI characteristics of different lesioning techniques.

## Data Availability

The data that support the findings of this study are available from the corresponding author, upon reasonable request.
